# The antimicrobial polymer PHMB enters cells and selectively condenses bacterial chromosomes

**DOI:** 10.1038/srep23121

**Published:** 2016-03-21

**Authors:** Kantaraja Chindera, Manohar Mahato, Ashwani Kumar Sharma, Harry Horsley, Klaudia Kloc-Muniak, Nor Fadhilah Kamaruzzaman, Satish Kumar, Alexander McFarlane, Jem Stach, Thomas Bentin, Liam Good

**Affiliations:** 1 Department of Pathology and Pathogen Biology, Royal Veterinary College, University of London, Royal College Street, London, NW1 0TU, UK; 2 Tecrea Ltd, London Bioscience Innovation Centre, 2 Royal College Street, London, NW1 0NH, UK; 3 Nucleic Acids Research Laboratory, CSIR-Institute of Genomics and Integrative Biology, Mall Road, Delhi-110 007, India; 4 Centre for Clinical Science & Technology, University College London, Wolfson House, 2–10 Stephenson Way, London NW1 2HE, UK; 5 Faculty of Veterinary Medicine, Universiti Malaysia Kelantan, Locked bag 36, Pengkalan Chepa, 16100 Kota Bharu, Kelantan, Malaysia; 6 Division of Animal Biotechnology, Indian Veterinary Research Institute, Izatnagar, Bareilly, Uttar Pradesh, 243 122, India; 7 School of Biology, University of Newcastle, Newcastle upon Tyne, NE1 7RU, UK; 8 Department of Cellular and Molecular Medicine, University of Copenhagen, Blegdamsvej 3C, 2200 Copenhagen N, Denmark

## Abstract

To combat infection and antimicrobial resistance, it is helpful to elucidate drug mechanism(s) of action. Here we examined how the widely used antimicrobial polyhexamethylene biguanide (PHMB) kills bacteria selectively over host cells. Contrary to the accepted model of microbial membrane disruption by PHMB, we observed cell entry into a range of bacterial species, and treated bacteria displayed cell division arrest and chromosome condensation, suggesting DNA binding as an alternative antimicrobial mechanism. A DNA-level mechanism was confirmed by observations that PHMB formed nanoparticles when mixed with isolated bacterial chromosomal DNA and its effects on growth were suppressed by pairwise combination with the DNA binding ligand Hoechst 33258. PHMB also entered mammalian cells, but was trapped within endosomes and excluded from nuclei. Therefore, PHMB displays differential access to bacterial and mammalian cellular DNA and selectively binds and condenses bacterial chromosomes. Because acquired resistance to PHMB has not been reported, selective chromosome condensation provides an unanticipated paradigm for antimicrobial action that may not succumb to resistance.

The broad-spectrum antimicrobial biocide polyhexamethylene biguanide (PHMB; polyhexanide) kills bacteria, fungi, parasites and certain viruses with a high therapeutic index^1^; it is widely used in clinics, homes and industry^2^ (Supplementary Table 1). It is most commonly used as a biocide, but is also an important drug used in several topical applications. PHMB is composed of repeating basic biguanidine units connected by hexamethylene hydrocarbon chains, providing a cationic and amphipathic structure. Despite extensive use over several decades, and efforts to identify acquired resistant mutants, resistance to PHMB has not been reported^3^. The evidence for a lack of acquired resistance is necessarily negative, and the possibility of mutation to resistance remains; nevertheless, it is striking that bacteria with acquired resistance have not been identified following extensive and varied usage.

The bactericidal properties of PHMB have been demonstrated against a range of species^2^,^4^, follow first order kinetics^5^ and have been observed within one hour at concentrations below 10 μg/mL^4^. Also, its high therapeutic index has long been attributed to the polymer having comparatively less activity against mammalian membranes^6^,^7^,^8^,^9^,^10^. The prevailing model for PHMB’s microbe-selective toxicity holds that PHMB disrupts microbial membranes preferentially. However, this model relies on data from artificial membrane studies and it does not explain how PHMB is able to kill diverse microbes, which differ in cell barrier composition^11^,^12^, nor does it explain observations that PHMB can induce DNA repair pathways^13^. Therefore, the literature contains conflicting evidence and interpretations regarding the antibacterial mechanism of action of PHMB. When considering the membrane disruption model and possible alternatives, it may be important to recognize that PHMB has a capacity for both electrostatic and H-bonding interactions^14^, which could occur at many possible targets in cells. For example, PHMB binding to nucleic acid has been demonstrated *in vitro*^15^, raising at least one possible alternative mechanism of action.

To re-examine its mechanism(s) of action, we subjected PHMB to cellular, molecular and biophysical analysis, using both bacterial and mammalian cell systems. We examined its membrane activities and cellular effects using a PHMB-fluorophore conjugate together with cell growth, microscopy and physiological assays. Also, we examined PHMB/nucleic acid interactions using biophysical methods. Surprisingly, the results reveal that PHMB enters both bacterial and mammalian cells, condenses bacterial chromosomes and is excluded from mammalian nuclei. The outcome suggests a new model to explain its selective antimicrobial activities.

## Results

### Bacterial cell membrane activities of PHMB

**Figure 1:**
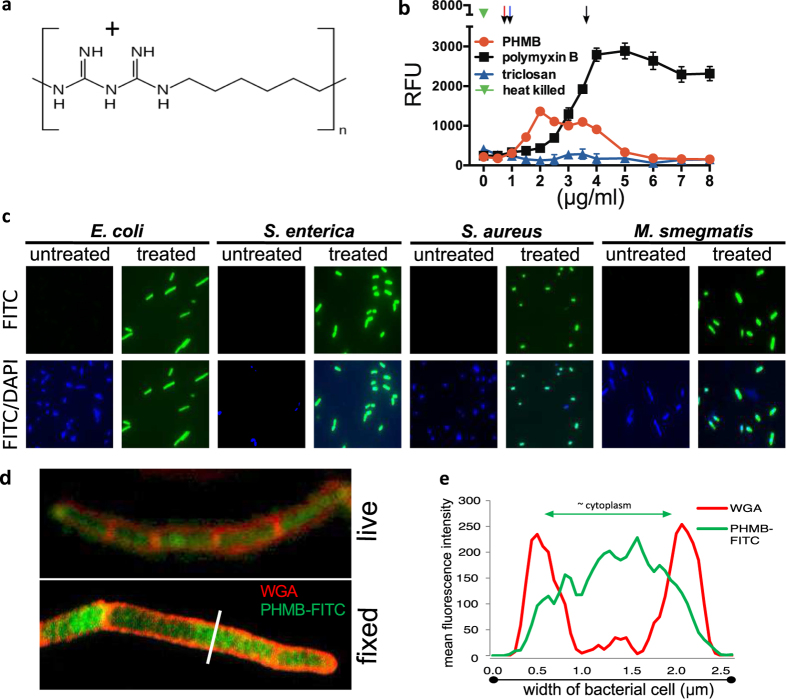
PHMB effects on cell membrane permeability and entry into bacteria. (**a**) Structure of polyhexamethylene biguanide (PHMB; CAS# 27083-27-8); alternative chemical names: polyhexanide; example trade names: Vantocil™, Cosmocil™, Baquacil™, Prontosan^®^. See Supplementary Table 1 for further details. PHMB is composed of repeating basic biguanidine units connected by hexamethylene hydrocarbon chains, providing a cationic and amphipathic structure with a high capacity for hydrogen bonding, electrostatic and hydrophobic interactions. PHMB preparations typically comprise polymers of mixed length with amine, guanidine and cyanoguanidine end groups (eg. average n = 13.8, 3,035 g/mol^44^) (**b**) Effects of PHMB, heat, polymyxin B (positive control) and triclosan (negative control) on cell permeability to SYTOX^®^Green. The MIC values for the antibacterials tested are indicated with colour-coded vertical arrows, at top. (**c**) Fluorescence microscopy of PHMB-FITC entry into diverse bacteria. PHMB-FITC (2 μg/mL) was added to bacterial cultures and the cells were counter stained with DAPI. (**d**) Confocal image showing localisation of PHMB-FITC (green) in *B. megaterium*; bacteria were counterstained with the membrane localising probe wheat germ agglutinin (WGA) conjugated to Alexa Fluor-555 (red) and visualized as live (top) and fixed (bottom) cells; Bar = 5 μm. (**e**) Fluorescence intensity profile plot analysis of cellular localisation of PHMB-FITC and WGA fluorescence (the white line indicated the cross section used for analysis). The green line indicates the levels of FITC and position (mainly within the cell). The red line indicates the levels of WGA and position (mainly within the membrane).

If the antibacterial activity of PHMB (Fig. 1a) is due to membrane disruption, as widely reported^6^,^7^,^8^,^9^,^10^, it would be expected to permeabilise bacterial cell barriers at growth inhibitory and sub growth inhibitory concentrations. To test this model, we first established the minimal inhibitory concentrations (MIC) and time kill properties for PHMB against *Escherichia coli (*strains K-12 and MG1655) and *Salmonella enterica* serovar Typhimurium (strain LT2). As reported previously^2^,^4^, PHMB displayed potent growth inhibitory and cidal properties (Supplementary Table 2 and Supplementary Fig. 1). Also, following treatment, we examined cells using light microscopy. Unexpectedly, growth inhibitory concentrations of PHMB did not lyse cells, as monitored by bright-field microscopy. To assess cell barrier damage that could be invisible to microscopy, *E. coli* K-12 cultures were grown to mid-log phase, treated with PHMB in the presence of the fluorescent membrane integrity probe SYTOX^®Green^, and then monitored using fluorimetry. This probe is useful as an indicator of membrane damage; because it is normally excluded from intact bacteria and its fluorescence quantum yield increases upon DNA binding. Therefore, intact bacteria are expected to display low fluorescence, and fluorescence is expected to increase following cell barrier damage^16^. As anticipated, freshly grown *E. coli* cultures displayed large increases in fluorescence following treatment with the known cell wall disruptor polymyxin B or heat treatment (Fig. 1b). Unexpectedly, PHMB treatment resulted in comparatively lower levels of fluorescence. Most strikingly, higher concentrations of PHMB resulted in fluorescence at background levels. These observations are not compatible with membrane disruption as the main antibacterial mechanism, and therefore raised further doubt about the established model.

### PHMB enters bacteria

If PHMB’s primary target is not bacterial cell barriers, or not exclusively cell barriers, then it likely acts internally, and this would require cell entry. To test for bacterial entry, we synthesised a PHMB-FITC conjugate (Supplementary Fig. 2a,b) and assessed uptake into Gram-positive (*Staphylococcus aureus*), Gram-negative (*Escherichia coli* and *Salmonella enterica* serovar Typhimurium) and acid-fast (*Mycobacterium smegmatis*) bacteria using microscopy and flow cytometry (Fig. 1c, Supplementary Fig. 3). Strong cell-associated green fluorescence was observed in all species tested (Fig. 1c). To examine cell localisation more thoroughly, the large-sized bacterium *Bacillus megaterium* was treated with PHMB-FITC, counter-stained with membrane localizing wheat germ agglutinin (WGA-red), and examined by fluorescence microscopy (Fig. 1d). Cell entry was observed in both live and fixed cells, and a fluorescence intensity profile analysis shows that PHMB-FITC localized within the cytoplasm, without accumulation at the cell barrier (Fig. 1e).

The observation that PHMB enters cells at low microgram/mL concentrations suggests that it may enter live cells. To investigate whether PHMB uptake into bacteria requires energy metabolism, mid-log phase *E. coli* cultures were incubated at 37 °C, or at 4 °C for 2 hours to reduce cellular ATP levels. Subsequently, cells were treated with PHMB-FITC (0–6 μg/ml) and further incubated on ice for 2 hours. Cell-associated PHMB-FITC fluorescence was quantified by fluorimetry (Supplementary Fig. 3b). Cells held at 4 °C displayed reduced PHMB-FITC uptake relative to cells incubated at 37 °C, consistent with an energy dependent cell uptake process. Also, green fluorescent and motile bacteria were observed at several time points during PHMB treatment (Supplementary Fig. 3). Because bacterial motility is energy dependent^17^, the evidence indicates that PHMB-FITC enters metabolically active cells. Therefore, PHMB enters diverse bacteria and entry was observed in motile cells.

### PHMB arrests cell division and condenses bacterial chromosomes

**Figure 2:**
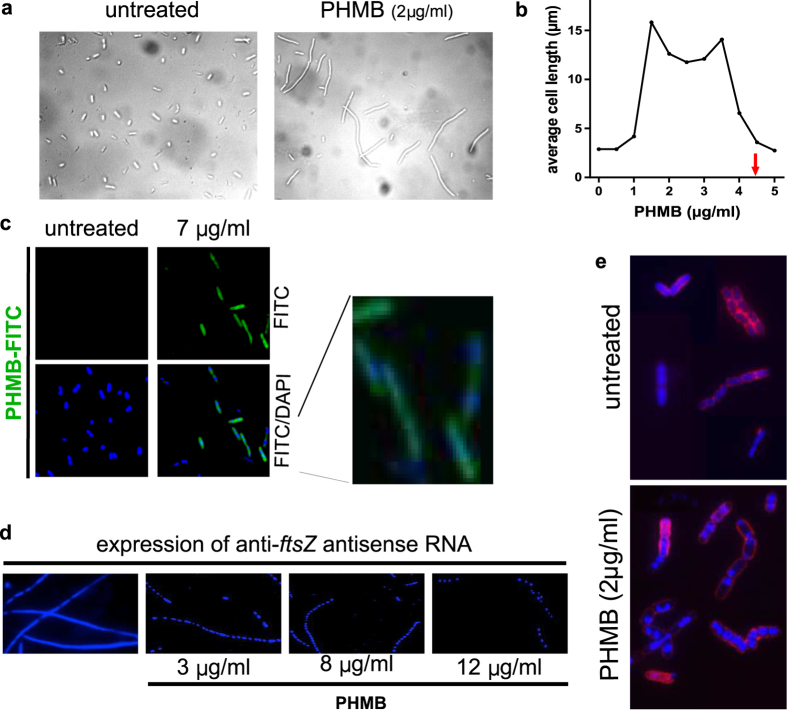
PHMB-mediated cell elongation and chromosome condensation in bacteria. (**a**) *E. coli* was treated with PHMB for 90 minutes and examined by bright field microscopy. (**b**) Mean cell length as a function of PHMB concentration. MIC (arrow) is indicated. (**c**) Pattern of chromosome distribution in cells following PHMB-FITC treatment. Cultures of *E. coli*, strain K-12 were treated with PHMB-FITC, counter stained with DAPI and examined using fluorescence microscopy. Chromosomes appear as condensed DAPI-stained foci; more apparent in the enlarged image. (**d**) Pattern of chromosome distribution in filamentous/multinucleated *E. coli* following PHMB exposure. RNA silencing of *ftsZ* expression was used to arrest cell division, and cells were then untreated or treated with PHMB, stained with DAPI and examined by fluorescence microscopy. (**e**) Pattern of chromosome distribution in *B. megaterium* cells that were untreated or treated with PHMB, stained with DAPI and WGA-red and examined using fluorescence microscopy.

When examining *E. coli* by microscopy, we noted that PHMB treated cells often displayed an elongated morphology, which can be characteristic of cell division inhibition (Fig. 2a). To measure the effects of PHMB on cell elongation, we titrated PHMB into growing cultures of *E. coli* strain SS996 (*vide infra*) and measured cell lengths. At growth inhibitory concentrations, more than 80% of cells elongated (Fig. 2b; Supplementary Table 2). Also, we observed that *E. coli* treated with growth inhibitory concentrations of PHMB or PHMB-FITC followed by DAPI-staining displayed blue fluorescent foci near the cell centre (Fig. 2c). These structures resembled nucleoids^18^. To ease visualisation of the DNA foci, we generated filamentous/multinucleated populations of *E. coli* by inhibiting cell division through RNA silencing of the essential cell division gene *ftsZ*^19^. RNA silencing was selected for this experiment because it enables specific and controllable repression of translation of essential gene transcripts^20^. Genes that are growth essential cannot be knocked-out by genome disruption methods, as this would result in non-viable strains. In the absence of PHMB, filamentous cells showed uniform DAPI staining, whereas PHMB treated cells displayed blue “strings of beads” (Fig. 2d). Similarly, in the large-sized Gram-positive bacteria *B. megaterium* we observed DAPI-stained foci following PHMB treatment (Fig. 2e). These results, in both Gram-negative and Gram-positive species, show that PHMB exposure leads to condensed chromosomes within bacteria.

### PHMB-mediated antibacterial effects are independent of stress response pathways

Cell elongation and chromosome condensation are characteristic morphologies that are often associated with the bacterial SOS response^21^,^22^. Therefore, we considered that these effects could involve this response. However, in the case of PHMB-mediated effects, an SOS response seemed unlikely. First, the SOS response is typically associated with DNA damage and there is no evidence for PHMB-mediated genotoxic or epigenetic effects^23^. Second, the condensation observed following *ftsZ* silencing and PHMB treatment occurred in a *recA*^−^ strain (TOP10), which is an SOS response mutant. Nevertheless, antimicrobial mechanisms are notoriously difficult to decipher and may involve multiple mechanisms. Therefore, we decided to assess the possible involvement of an SOS response and other stress response pathways using an SOS reporter strain and a panel of *E. coli* stress response pathway mutants.

**Figure 3:**
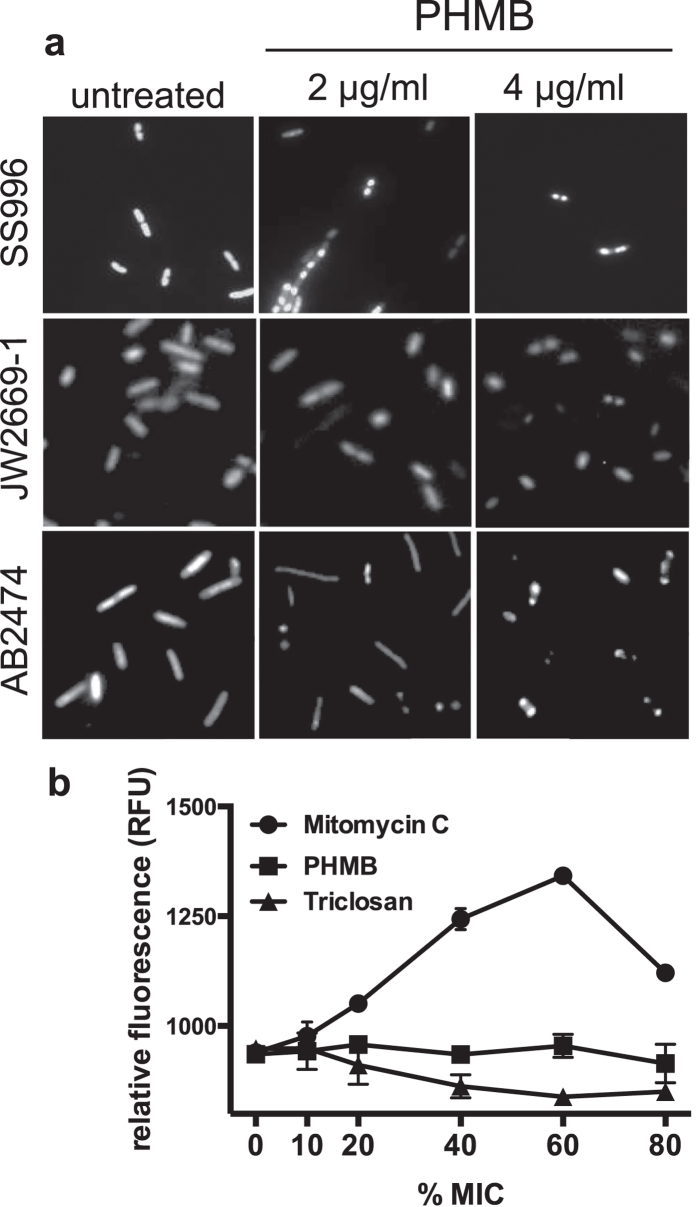
Effects of PHMB on bacterial SOS responses. (**a**) Chromosome condensation in *E. coli* strains SS996 (*sulB103*; FtsZ mutant insensitive to SulA), JW2669 (*recA*^−^; knock-out of *recA*) and AB2474 (*lexA1*, mutation that prevents SOS response induction) following treatment with PHMB for 2 hours. Cells were DAPI stained to reveal DNA. (**b**) SOS response reporter expression, quantified by fluorimetry. The SOS reporter *E. coli* strain SS996 carrying a chromosomal *sulAp-gfp* fusion was untreated or treated with PHMB, mitomycin C, a known SOS inducer, or triclosan, which does not induce an SOS response. The MIC values against SS996 were PHMB, 0.75 μg/mL; triclosan, 2 μg/mL; mitomycin C, 0.06 μg/mL, and these values were used to calculate %MIC.

To test whether PHMB-mediated effects on cell elongation and chromosome condensation are altered by mutations to the SOS response pathway, we evaluated morphological responses in three mutant *E. coli* strains. Strain SS996 is able to initiate an SOS response, but due to a *sulB* mutation the response does not lead to cell division inhibition. This is because SS996 has a mutant allele of *ftsZ* (*sulB*103), the product of which is insensitive to the action of the SOS-induced cell division inhibitor SulA^24^. Strain JW2669 does not produce functional RecA, and so is SOS deficient. Strain AB2474 has a mutation in the LexA repressor that renders it non-cleavable by RecA, and so is not able to induce an SOS response (additional strain details are given in Fig. 3 and Supplementary Information Table 3). Cultures in mid-log phase were treated with PHMB, DAPI stained and observed under a fluorescence microscope. As observed with *E. coli* K-12, the mutant strains displayed elongated morphologies and condensed chromosomes following PHMB treatment (Fig. 3a). Therefore, PHMB-mediated cell division and chromosome structure effects occur independently of an SOS programmed response.

To more directly measure whether PHMB induces an SOS response, we used the *E. coli* strain SS996, which is a reporter strain that contains a *sulAp-gfp* chromosomal SOS response/reporter system^24^,^25^. If an SOS response is caused by PHMB, then PHMB exposure should induce GFP expression in this strain. Cultures of SS996 were treated with PHMB for 18 hours and then green fluorescence was measured. Mitomycin C, which damages DNA, was included as a positive control, and triclosan, which inhibits fatty acid biosynthesis, was included as a negative control. As expected, mitomycin C induced a large increase in GFP expression and triclosan did not induce GFP expression. In contrast to mitomycin C, PHMB did not induce GFP expression, indicating that PHMB does not induce an SOS response (Fig. 3b).

We next tested whether strains with defective or deregulated SOS responses differ in susceptibility to PHMB. To test *recA*-mediated effects on susceptibility, we used a strain of *E. coli* that lacks *recA* (JW2669) and a strain that overexpresses *recA* upon addition of the inducer IPTG (ASKA JW2669), and determined MIC values. Neither deletion nor induced overexpression of *recA* altered susceptibility to PHMB (Supplementary Information Table 3, rows shaded in dark grey). In contrast, the *recA*^−^ strain was 2-fold more susceptible to the SOS response inducing drug nalidixic acid, and *recA* overexpression reduced susceptibility to nalidixic acid 8-fold. To test *lexA*-mediated effects on PHMB susceptibility, we used the *lexA1(Ind*^−^) strain AB2474, which is unable to induce an SOS response. Relative to the parent, AB2474 was 1-fold more susceptible to PHMB and 1-fold less susceptible to nalidixic acid (Supplementary Table 3, rows shaded in light grey). Therefore, none of the SOS response mutants tested displayed changes in susceptibility to PHMB that indicate involvement of an SOS response.

Finally, we considered whether other (non-SOS) stress response pathways affect susceptibility to PHMB. We tested a series of known *E. coli* stress response mutants in parallel with their parent for susceptibility to PHMB. None of the mutants displayed changes in MIC values that suggest a functional involvement of any of the stress response pathways (Supplementary Table 3). Therefore, the antibacterial effects of PHMB occur independently of the panel of stress response mechanisms tested.

### PHMB condenses bacterial chromosomes *in vitro*

**Figure 4:**
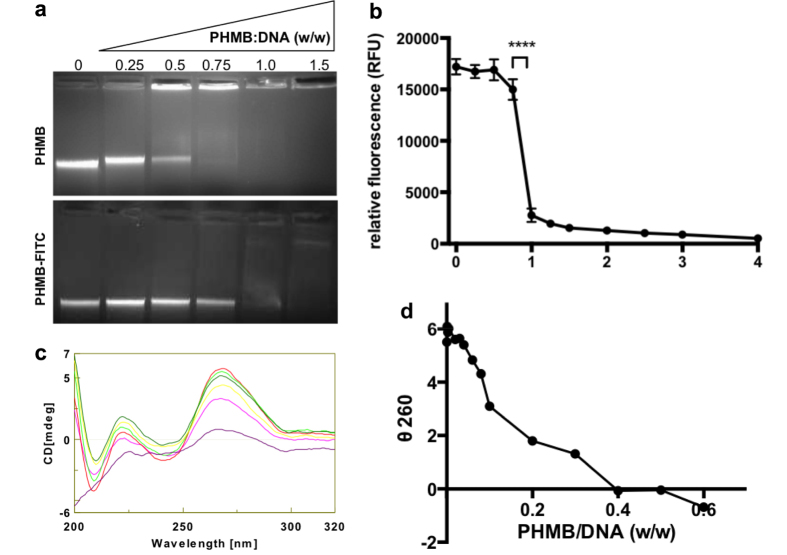
PHMB binding to bacterial chromosomal DNA *in vitro*. (**a**) PHMB or PHMB-FITC was mixed with isolated chromosomal DNA from *E. coli* strain K-12, and samples were analysed by EMSA. Patterns of retarded in-gel mobility indicate DNA binding by PHMB. (**b**) PHMB-mediated exclusion of SYTOX®Green binding to isolated *E. coli* chromosomal DNA, where reduced fluorescence indicates DNA binding by PHMB. (**c**) Circular dichroism spectroscopy of mixtures of PHMB and isolated *E. coli* chromosomal DNA. (**d**) Plot of ellipticity as a function of PHMB:DNA ratios.

If PHMB condenses bacterial chromosomes inside cells, this could occur *via* direct or indirect effects on DNA. We suspected direct effects, because PHMB has been shown to bind to DNA fragments *in vitro*^15^. We decided to examine the DNA binding properties of PHMB using isolated *E. coli* chromosomal DNA. PHMB-DNA interactions were first examined using an electrophoretic mobility shift assay (EMSA) and a dye exclusion assay. PHMB was mixed with chromosomal DNA isolated from *E. coli* K-12, and the mixtures were fractionated in agarose/TBE gels, followed by DNA staining with ethidium bromide. PHMB:DNA mixtures having wt:wt ratios of ≥0.5 displayed clearly retarded electrophoretic mobility, as indicated by retention of DNA in the well (Fig. 4a). Similar results were obtained for PHMB-FITC. Retarded mobility and retention in wells is consistent with stable interactions between PHMB and DNA. Also, the EMSA assays indicated reduced ethidium bromide fluorescence in the presence of PHMB or PHMB-FITC, suggesting that ethidium bromide was prevented from binding to DNA due to formation of PHMB:DNA complexes. This observation was further investigated using the DNA binding dye SYTOX^®^Green in a dye exclusion assay. In the absence of PHMB, SYTOX^®^Green bound isolated *E. coli* DNA, as indicated by a large increase in fluorescence, relative to the addition of dye alone. However, prior addition of PHMB reduced fluorescence >80% (Fig. 4b). Therefore PHMB forms complexes with bacterial DNA in a way that retards electrophoretic mobility and masks DNA access to DNA ligands. The results of each of these experiments indicate that PHMB binds directly to DNA.

To learn more about how PHMB binding to DNA impacts the structure of chomosomal DNA, we used biophysical methods and microscopy. Combinations of PHMB and isolated *E. coli* chromosomal DNA were examined by circular dichroism (CD) spectroscopy. PHMB alone did not show a characteristic CD spectrum, whereas isolated chromosomal DNA showed a typical DNA spectrum with a positive maximum ellipticity around 260 nm, a negative cross over at 252 nm and a negative trough at around 245 nm. This allowed us to assess changes in the CD spectrum of DNA following addition of PHMB. Mixtures of PHMB and DNA displayed reduced ellipticity at 260 nm, indicative of structural changes to the DNA upon PHMB binding (Fig. 4c,d). Also, dynamic light scattering (DLS) revealed that PHMB binding to DNA results in the formation of nanoparticles of approximately 50–60 nm, with a low polydispersity index (Supplementary Fig. 4a). Finally, transmission electron microscopy (TEM) and fluorescence microscopy also indicated that PHMB binding to DNA results in the formation of nanoparticles (Supplementary Fig. 4b,c). Therefore, these results confirm earlier reports that PHMB binds DNA^26^, and reveals that PHMB binds isolated bacterial chromosomal DNA and can condense chromosomes into a low polydispersity population of nanoparticles.

### The antibacterial effects of PHMB are suppressed by a dsDNA ligand

**Figure 5:**
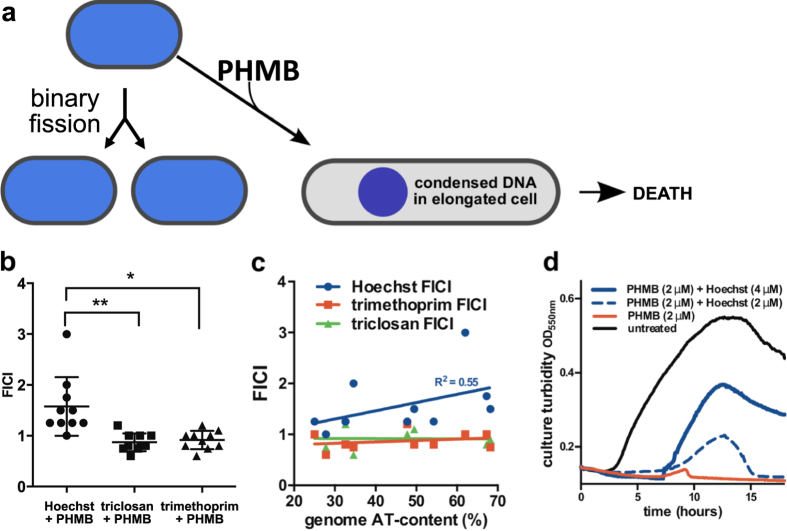
Model for the antibacterial mechanism of action of PHMB, and suppression of growth inhibition. (**a**) Proposed antibacterial mechanism of action of PHMB. (**b**) Pairwise growth inhibition interactions between PHMB and Hoechst 33258 and negative control non-DNA-binding ligands (triclosan and trimethoprim) in diverse bacterial species. (**c**) Relationship between bacterial genome AT-content and antibacterial interactions with PHMB. Plot of growth inhibition interactions and DNA AT-content in diverse species. Interaction values are fractional inhibitory concentration indicies (FICI) between PHMB and Hoechst 33258 or negative control non-DNA-binding ligands (trimethoprim and triclosan). (**d**) *Bacillus megaterium* growth inhibition by PHMB and suppression by combinations with the DNA ligand Hoechst 33258 (blue lines). See Supplementary Table 4 for species list and inhibition values.

Our results for PHMB effects on bacteria cannot be reconciled with the membrane disruption model for PHMB’s primary antibacterial mechanism of action. Rather we propose a new model, where PHMB enters bacteria and then condenses chromosomes, as illustrated in Fig. 5a. If correct, the new model would also predict functional interactions between PHMB and other DNA ligands, and this idea provided us with a way to test the model. In brief, if this model were correct, small molecular weight DNA ligands would be expected to suppress PHMB’s antibacterial potency by competing for DNA binding sites within chromosomes. To test this possibility, pairwise combinations of PHMB and Hoechst 33258 were used in growth susceptibility assays. Hoechst 33258 is a DNA ligand that binds preferentially to the minor groove of AT-rich sequences^27^ and it is cell-permeable, making it a suitable choice for this competition experiment.

Drug interactions were calculated as fractional inhibitory concentration index values (FICI) using a panel of diverged bacterial species. The FICI values for PHMB:Hoechst were significantly higher than for PHMB combined with either of two non-DNA ligand antibacterials (Fig. 5b). Also, the FICI values for PHMB:Hoechst combinations show a positive correlation with chromosome AT-content (Fig. 5c). In other words, PHMB’s antimicrobial effects depend on access to DNA inside cells. In *B. megaterium*, the effects of PHMB:Hoechst combinations were striking, where growth inhibition by PHMB was suppressed using subinhibitory Hoeschst 33258 concentrations (Fig. 5d). Therefore, the small molecule DNA ligand Hoechst 33258 rescued bacteria from inhibitory concentrations of PHMB.

These pairwise drug interactions reveal that the antibacterial effects of PHMB occur mainly *via* targeting DNA in bacteria. Consistent with the cell permeability change profile observed for PHMB (Fig. 1b); the results also indicate competition between PHMB and a DNA ligand for DNA binding sites inside cells. Therefore, the results from separate experiments involving two known DNA ligands are consistent with our new model for PHMB activity.

### PHMB enters mammalian cells but is excluded from nuclei

**Figure 6:**
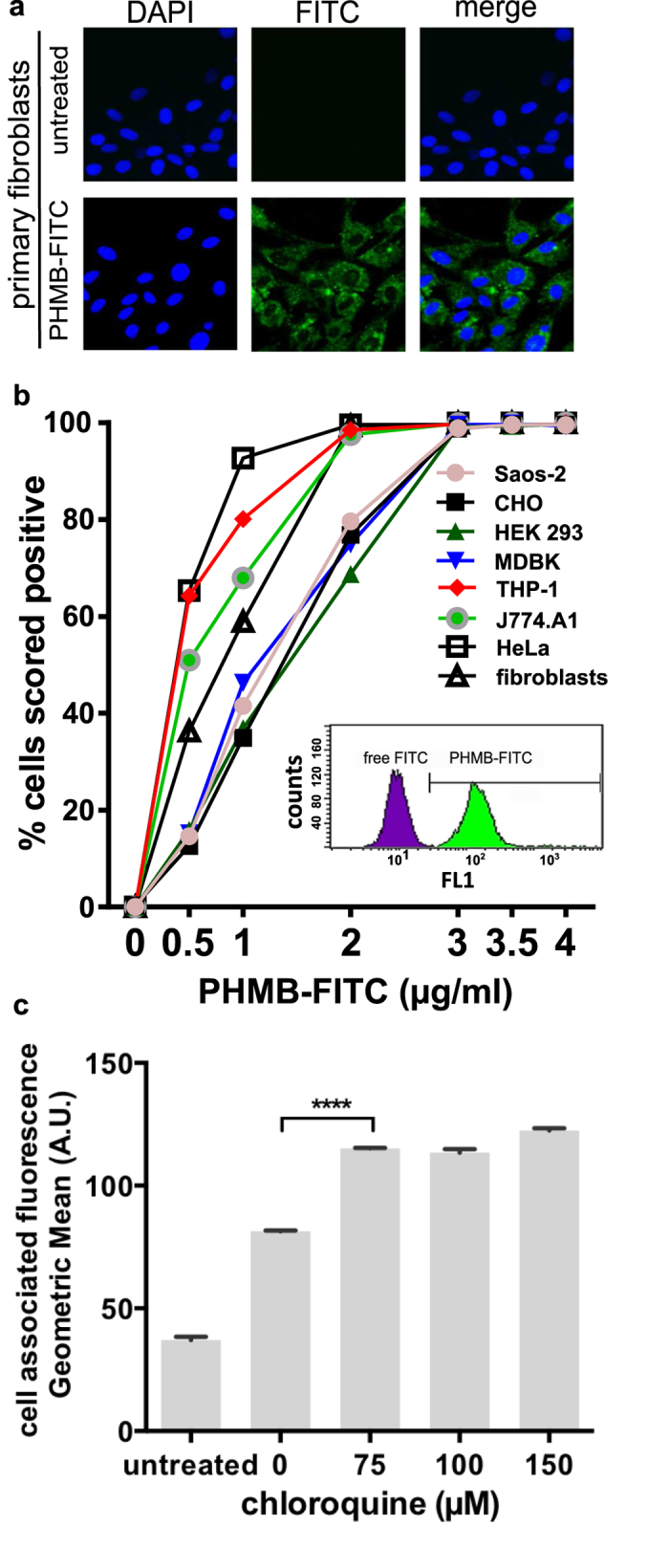
PHMB entry into mammalian cells. (**a**) Primary fibroblasts were treated with PHMB-FITC (3.5 μg/mL), counter stained with Hoechst 33258 and observed by fluorescence microscopy. (**b**) Flow cytometry analysis of a panel of mammalian cells treated with PHMB-FITC. Inset: a representative example of a flow cytometry histogram of HeLa cell populations that were untreated (purple population) or treated with PHMB-FITC (0.4 μg/mL) (green population). (**c**) HeLa cells were treated with PHMB-FITC (3.5 μg/mL) and chloroquine (0–20 μM) for 2 hours. The effects of chloroquine on fluorescence were measured by flow cytometry, using geometric mean fluorescence intensity (arbitrary units (A.U.), log scale).

The prevailing model for PHMB activity holds that PHMB kills bacteria through bacterial membrane damage and the polymer does not interact with mammalian cell membranes (see above). However, given the unexpected bacterial cell entry properties of PHMB, and our recent observations that PHMB enters cultured macrophage^28^ and keratinocytes^29^, we decided to directly assess its ability to enter a panel of mammalian cells. PHMB-FITC was added to several mammalian cell lines and primary fibroblasts and uptake was assessed by fluorescence microscopy and flow cytometry. We observed clear uptake into all cell types tested (Fig. 6a,b). Also, these conditions did not lead to disruption of mammalian cell membrane integrity (Supplementary Fig. 5a). Close inspection of the microscopy images reveals that PHMB-FITC was contained within vesicles and excluded from nuclei (Fig. 6a). If it is true that endosomes entrap PHMB, then release into the cytoplasm should de-quench PHMB-FITC and lead to an increase in fluorescence. This is because FITC fluorescence is quenched at low pH and the late endosomal pH is <6^30^, whereas the cytoplasmic pH is 7.4. We observed that the addition of chloroquine, an osmolytic/buffering agent^31^, increased the fluorescence of PHMB-FITC treated cells (Fig. 6c), consistent with polymer entrapment within endosomes. Therefore, PHMB efficiently enters mammalian cells, but is entrapped within endosomes, which appears to restrict entry into nuclei.

## Discussion

The arsenal of registered antimicrobial agents (antibiotics + biocides) exploits only a small number of defined mechanisms^32^. Past studies on the mechanism of action of PHMB focused on its ability to interact with microbial membranes in preference to mammalian membranes. The present study, in contrast, suggests an alternative model. The results demonstrate that PHMB is able to enter bacterial cells, arrest cell division and condense chromosomes, resulting in intracellular foci of DNA. PHMB is revealed as the first example of a drug that binds and condenses bacterial chromosomes. Indeed, this is the first example of any drug that condenses chromosomes.

Chromosome condensation has not previously been considered as an antibacterial mechanism of action; however, the feasibility of using a cationic polymer to directly condense chromosomes in bacteria is suggested by several prior findings: (*i*) the regulation of bacterial DNA condensation and decondensation appears to involve polyamine and basic protein binding^33^, (*ii*) condensation aids chromosome partitioning during cell division^34^, (*iii*) overexpression of histone-like proteins leads to nucleoid condensation and bacterial cell death^35^,^36^, (*vi*) chromosomes in bacterial cell lysates are condensed by the addition of polylysine^37^, and (*v*) certain cationic polymers enter bacteria^38^,^39^. Therefore, while indirect mechanisms leading to chromosome condensation in bacteria remain formally possible, our data indicates a direct mechanism, where PHMB binds and condenses DNA following cell entry (Fig. 5a), and the prior evidence outlined above is consistent with this new model.

A chromosome condensation model for the antibacterial action of PHMB (Fig. 5a) can explain how PHMB kills bacteria, but also raises a new and difficult question. How can chromosome condensation provide a selective antibacterial mechanism, given that all organisms have chromosomes? In other words, if PHMB enters cells and condenses bacterial chromosomes, why doesn’t it also kill mammalian cells and display toxic effects when used in clinical applications? The data shown in Fig. 6 provide an answer by showing that PHMB’s distribution within mammalian cells is partitioned. Specifically, it localizes within endosomes and is excluded from nuclei. Therefore, PHMB’s antibacterial selectivity appears to involve differential target access through drug partitioning inside cells, rather than by the well-established principles of target recognition and structure conservation^40^.

Mammalian cell uptake and nuclear exclusion of PHMB are unexpected observations; however, they may reflect aspects of how mammalian cells evolved together with microbes. For example, cationic antimicrobial peptides are central to innate immunity, and our observations may indicate mechanisms that protect host cells against endogenous cationic antimicrobial peptides and other natural molecules that could bind cellular DNA. Also, these observations raise interesting questions about potential toxicity and how PHMB kills eukaryotic pathogens. Recently, we reported that PHMB is able to enter the parasite *Leishmaniasis major*, and following parasite cell entry, PHMB disrupts chromosomes within nuclei^28^. Therefore, we have observed similar mechanisms in both bacteria and parasites, suggesting that chromosome condensation or disruption appears to be a major mechanism of action of PHMB that is effective against diverse pathogens. Understanding antimicrobial mechanisms can aid further drug development^41^. We hope that the results of this study will inspire antimicrobial strategies designed to selectively condense microbial chromosomes - a mechanism that does not appear to succumb to acquired resistance^3^.

## Materials and Methods

### Synthesis of PHMB–FITC conjugates

Polyhexamethylene biguanide (PHMB) was from Arch Chemicals (UK) and Tecrea Ltd, (UK). Fluorescein isothiocyanate (FITC, 2 mg, Sigma-Aldrich, UK) was dissolved in 800 μl dimethyl formamide (Sigma-Aldrich, UK), containing 50 μl of N,N-diisopropylethylamine (Sigma-Aldrich, UK). The mixture was combined with 200 μl aqueous PHMB (50 mg), and shaken overnight at room temperature. The resulting solution was dialyzed using a molecular weight cut off membrane 3.5 kDa against 50% aqueous ethanol for 5 days with intermittent change of dialysate (10 times, 500 mL), lyophilised to obtain fluoresceinyl-PHMB (PHMB-FITC) and characterized by infrared spectroscopy, IR (Nujol), ν (cm^−1^): 756 cm^−1^ (C = S stretching).

### Determination of Minimal Inhibitory Concentrations (MIC), Minimal Bactericidal Concentrations (MBC) and Fractional Inhibitory Concentration Index (FICI)

All *E. coli* strains, *S. enterica* serovar Typhimurium LT2 and *S. aureus* NCTC 6571 were grown in Mueller Hinton broth (MHB, Fluka, Germany) at 37 °C overnight. Where required, MHB was supplemented with kanamycin or chloramphenicol/IPTG (Supplementary Table 3). MICs were determined by serial dilution of the antibacterial in 200 μl MHB containing 10^5^ colony forming units (CFU)/mL using 96-well plates (Costar, UK). Plates were incubated for 18 hours at 37 °C in a BioTek Power-Wave X340I spectrophotometer with shaking for 5 seconds every 5 minutes followed by recording of the absorbance at 550 nm. The MIC was scored as the lowest concentration of compound at which no growth was observed. To determine the minimal bactericidal concentration (MBC; >10^3^ CFU/mL reduction), bacterial cultures at 10^5^ CFU/mL were treated or not treated with PHMB and at specific time points samples were diluted and plated on LB agar plates. CFUs were counted after 18 hours of incubation at 37 °C. FICIs were determined using the checkerboard assay as described 42, using Hoechst 33258 (Sigma-Aldrich, UK).

### Bacteria cell membrane permeability assay

*E. coli* K-12 from mid log phase (10 μl of culture, OD_600_ adjusted to 0.1) were transferred to 96-well plates containing PHMB, polymyxin B or triclosan (0–8 μg/mL) in 100 μl phosphate buffered saline (PBS), and incubated at 37 °C for 60 minutes in a BioTek Power-Wave X340I spectrophotometer with shaking for 5 seconds every 5 minutes. To generate cells with maximum permeability to SYTOX^®^Green, untreated cultures were incubated for 10 minutes in a heating block maintained at 70 °C. The dye SYTOX^®^Green (Invitrogen, UK) was added to a final concentration of 1 μM, and changes in fluorescence emission were monitored at 535 nm upon excitation at 485 nm using a Wallac Victor 1420 Multi label counter (PerkinElmer, UK). SYTOX^®^Green fluoresces strongly upon binding to DNA, and fluorescence was taken as an indication of membrane permeabilisation^43^.

### Epi-fluorescence microscopy of bacteria and particles

Overnight bacterial cultures of *E. coli* K-12, SSPP6 and JW2669-1, *S. enterica* serovar Typhimurium LT2, and *S. aureus* NCTC 6571 were diluted 1:50 in MHB and incubated for 2 hours to reach mid log phase. *M. smegmatis* MC2155 was grown in Middlebrook 7H9 broth with 10% OADC enrichment (BD Biosciences, UK) for 18 hours. Aliquots from mid log phase cultures were diluted in MHB (*E. coli* and *S. enterica*) or 7H9 broth (*M. smegmatis*) to 0.1 OD_600_, and 100 μl aliquots were transferred to 1.5 mL tubes, PHMB-FITC (0–8 μg/mL) was added and the cultures were incubated for 90 minutes. Following incubation, bacteria were washed 3 times using 200 μl 1× PBS via centrifugation at 5000 rpm for 5 minute and removal of the supernatants. Bacteria pellets were resuspended in 100 μl of 1 μM DAPI (Invitrogen, UK) in 1× PBS and incubated at room temperature for 10 minutes, then mounted on a glass slide and observed under a fluorescence microscope (Leica DM4000B microscope with Zeiss “AxioVision” software).

For particle analyses, PHMB:DNA mixtures were prepared as described above for the dye exclusion assay and loaded on an agarose bed (1.5% in 1× PBS) prepared on a glass slide, overlaid with a glass coverslip and observed under an upright fluorescence microscope using a FITC filter set (400× magnification, Leica DM4000B microscope, “AxioVision” software).

### Electrophoretic mobility shift assay (EMSA)

Chromosomal DNA from overnight cultures of *E. coli* K-12 was isolated using GenElute™ bacterial chromosomal DNA isolation Kit (Sigma-Aldrich, UK) according to the manufacturer’s instructions. Mixtures of isolated chromosomal DNA and PHMB were prepared by titrating 0.5 μg of chromosomal DNA with PHMB (0–0.75 μg) in a final volume of 50 μl 1× PBS followed by incubation at 37 °C for 30 minutes. The resulting samples were combined with 6× Blue/Orange loading dye (Promega, UK) and analyzed by electrophoresis using 0.8% agarose gels containing ethidium bromide.

### Circular dichroism (CD) spectroscopy

CD spectra of *E. coli* DNA (0.1 mg/mL) from *E. coli* K-12 were recorded from 190 to 320 nm in 10 mM phosphate buffer, pH 7.0 at 25 °C in a CD spectropolarimeter (JASCO, J-810 model, Japan) using a 0.1 cm path length cuvette. Six scans (20 nm/minute) were taken and the results were averaged. PHMB:DNA interactions were monitored by titrating isolated chromosomal DNA (150 μl, 100 μg/mL) with PHMB (0.0025–100 μg/mL, final volume). A CD spectrum base line correction was made for each PHMB concentration. Structural changes in DNA were monitored by plotting ellipticity at 260 nm (θ_260_) against PHMB concentration. The experiments were repeated three times.

### Dye exclusion assay

PHMB (0–4 μg/mL, final) and isolated chromosomal DNA from *E. coli* K-12 (1 μg/mL, final) were mixed in 100 μl 1× PBS in a 96-well plate and incubated at 37 °C for 30 minutes. Following incubation, SYTOX^®^ Green was added to 100 nM, and the plates were further incubated for 10 minutes at 37 °C and fluorescence was measured as described in the bacteria cell membrane permeability assay; see above.

### Confocal microscopy of bacteria

To explore the spatial distribution of PHMB in live bacteria, one of the largest known Gram positive bacteria, *B. megaterium*, was exposed to PHMB-FITC and counterstained with a fluorescent membrane marker. *B. megaterium* was cultured in LB broth overnight before dilution to 10^8^ CFU/mL in fresh broth containing a final concentration of 5 μg/mL of PHMB-FITC. Following incubation in a shaker for 120 minutes at 37 °C, the bacteria were washed and stained with DAPI as described above. Bacterial pellets were re-suspended in 1× PBS and 80 μl of the suspension was centrifuged in a Shandon Cytospin 2 cytocentrifuge at 800 rpm (75 × g) for 5 minutes before circumscribing the slide-deposited bacteria with a hydrophobic barrier pen (ImmEdge pen, Vector Laboratories). Cells were fixed in 4% formaldehyde (Thermo Scientific, Fisher Scientific) in 1× PBS at room temperature for 15 minutes. The formaldehyde was aspirated and the preparation were washed three times with 1× PBS at 5 minute intervals. To label the bacterial cell membrane, the fixed bacteria were immersed in 100 μl of Hank’s balanced salt solution (HBSS) minus phenol red (Invitrogen) containing 5 μg/mL wheat germ agglutinin (WGA) conjugated to Alexa Fluor-555 (Invitrogen). Following 20 minute incubation at room temperature, the labeling solution was removed and the slides were washed twice at 5 minute intervals with fresh HBSS. The preparation was mounted with FluorSave reagent (Calbiochem) and a coverslip fixed in place with clear nail varnish. Slides were kept at −20 °C. Laser scanning confocal microscopy was conducted using a Leica SP5 microscope (Leica Application Suite, Advanced Fluorescence 3.1.0 build 8587 Software). Sequential scan Z-stacks (115 slice 1024 × 1024) were compiled at a line average of 96. ImageJ 1.49r was used to produce, analyse and profile plot dual channel composite Z-stacks.

### Generation of filamented bacteria

Overnight cultures of *E. coli* TOP 10 carrying a plasmid expressing *lac* promoter driven antisense RNA against bacterial cell division *ftsZ* mRNA (anti-*ftsZ*) were grown in MHB for 18 hours in the presence of chloramphenicol (30 μg/mL, Sigma-Aldrich, UK) to maintain the plasmid^19^. Overnight cultures were diluted 50-fold in MHB and IPTG (75 μm) was added to induce anti-*ftsZ* expression. Cultures were further incubated for 90 minutes in an orbital shaker at 37 °C in the presence or absence of PHMB. Following incubation, bacteria were washed and stained with DAPI as described above and observed under a fluorescence microscope (630× magnification, Leica DM4000B microscope, Zeiss “AxioVision” software).

### SOS response assay

Overnight cultures of *E. coli* strain SS996 (10^5^ CFU/mL) were sub-cultured in the absence or presence of PHMB, mitomycin C (Roche, UK) or triclosan (Ciba AG, Switzerland) in MHB for 18 hours in a 96-well plate in a Bio-Tek PowerWave X340I spectrophotometer as described above, and SOS response was assessed as described previously^25^. GFP expression was measured in a Wallac Victor^2^ 1420 Multi label counter using 485 nm (excitation) and 535 nm (emission).

### Dynamic light scattering

PHMB:DNA mixtures (25 μg/mL:10 μg/mL) were diluted with 900 μl of 0.2 μm filtered water and mean particle size was measured by dynamic light scattering in a Zetasizer Nano ZS instrument (Malvern instruments, UK). Mean size represents the average of 20 readings. Experiments were repeated independently three times.

### Transmission electron microscopy

PHMB:DNA (25 μg/mL:10 μg/mL) mixtures were loaded onto carbon coated copper grids, stained with aqueous 1% uranyl acetate for 30 seconds, air dried and visualised under a transmission electron microscope operated at 200 kV and 6500× magnification (Tecnai G2 30 U-twin, USA). Corresponding concentrations of PHMB alone were used as control.

### Mammalian cell culture

HeLa, HEK 293, MDBK, equine primary fibroblasts, Saos-2, CHO, and J774.A1 cells were maintained in DMEM (Invitrogen) with 10% FBS (Invitrogen), Penicillin (100 units/mL, Invitrogen) and streptomycin (100 μg/mL, Invitrogen). THP-1 monocytes were maintained in RPMI 1640 medium with 10% FBS. To sub-culture adherent cells, the growth medium was removed and cells were washed twice with Hank’s buffered salt solution (Invitrogen, UK). To detach cells, 2 mL of 0.05% trypsin - EDTA (Invitrogen, UK) was added and the cells were incubated at 37 °C for 5 minutes. Detached cells were counted using 0.4% Trypan blue (Invitrogen, UK) and the cells were seeded into 12 well tissue culture plates (0.1 × 10^6^ cells/well) and 75 cm^2^ tissue culture flask.

### Epi-fluorescence microscopy of mammalian cells

Equine primary fibroblasts were grown to ∼60% confluence and treated with PHMB-FITC (0–4 μg/mL) or free FITC (0.389 μg /mL; 1 μM) in DMEM and incubated for 2 hours. Following incubation, nuclei were stained using 1 μM Hoechst 33342 (Invitrogen, UK) in DMEM by incubating cells at 37 °C for 30 minutes. Following nuclear staining, cells were washed 3 times with PBS, and the extracellular fluorescence was quenched with 0.04% trypan blue (Invitrogen, UK) in ice cold 1× PBS for 10 minutes. Cells were washed twice with 1× PBS, mounted on glass slides with aqueous fluoromount (Sigma-Aldrich, UK) and observed under a fluorescence microscope (Leica DM4000B, “AxioVision” software). PHMB-FITC uptake was scored by flow cytometry using an FL1 filter set (FACSCalibur^TM^, CellQuest™ software, BD Bioscience). Similarly, PHMB uptake was assessed in other adherent cell lines (HEK 293, MDBK, Saos-2, CHO and J774.A1). To investigate uptake into suspension cells, 5 × 10^5^ THP-1 monocytes were transferred to a 96-well plate containing PHMB-FITC (0–4 μg/mL) in a final volume of 100 μl RPMI 1640 and incubated at 37 °C for 2 hours. Following incubation, cells were rinsed twice with 1× PBS and cellular uptake was quantified by flow cytometry. Free FITC (1 μM) and untreated cells were used as negative controls to set background fluorescence.

### Flow cytometry

Cell associated fluorescence of PHMB-FITC treated or untreated cells was analysed by flow cytometry (FACSCalibur^TM^; CellQuest™ software, BD Bioscience) using an FL1 filter set. Adherent mammalian cells were trypsinised and diluted to 10^6^ cells/mL prior to flow cytometry. Untreated cells were used to establish thresholds for gating fluorescence negative cells. Results were analysed using Flowjo7.6.5 software.

### Propidium idodide (PI) mammalian cell membrane integrity assay

To assess membrane permeability, HeLa cells were grown in 12-well plates and treated with PHMB (0–4 μg/mL) in DMEM for 2 hours. Following incubation, cells were rinsed twice with 1× PBS and treated with propidium iodide (PI, 2 μg/mL, Sigma-Aldrich, UK), for 15 minutes, PI uptake was analysed by flow cytometry.

### Endosome release assay

PHMB-FITC (3.5 μg/mL) was suspended in 1× PBS solutions in the pH range 4.06–7.4 (pH adjusted with 0.1 M HCl). The solutions were incubated at room temperature for 10 minutes and fluorescence was measured by recording emission at 535 nm upon excitation at 485 nm in a fluorimeter. HeLa cells were co-treated with 3.5 μg/mL PHMB-FITC and chloroquine (0–150 μM, Sigma-Aldrich, UK) for 2 hours at 37 °C and fluorescence was quantified by flow cytometry. The number of cells positive for uptake and the geometric mean (GM) was recorded to assess the cell-associated fluorescence from three independent experiments.

### Data analysis

Data are represented as mean ± standard deviation from at least three independent experiments. Data in Fig. 5 and Supplementary Fig. 5 were analysed by one-way ANOVA and data in Supplementary Fig. 2 were analysed by two-way ANOVA, followed by Tukey’s Post test (Prism 6, GraphPad, Inc., San Diego, CA). *P* < 0.05 was considered as statistically significant (*).

## Additional Information

**How to cite this article**: Chindera, K. *et al.* The antimicrobial polymer PHMB enters cells and selectively condenses bacterial chromosomes. *Sci. Rep.* **6**, 23121; doi: 10.1038/srep23121 (2016).

## Supplementary Material

Supplementary Information
