# Supplementary material for: The antimicrobial polymer PHMB enters cells and selectively condenses bacterial chromosomes
Source: Sci Rep. 2016 Mar 21;6:23121. doi: 10.1038/srep23121 (PMC4800398; doi:10.1038/srep23121)
Supplement: Supplementary Information [file srep23121-s1.pdf]

Supplementary Information

Title: The antimicrobial polymer PHMB enters cells and selectively condenses bacterial chromosomes

Authors: Kantaraja Chindera[1], Manohar Mahato[3], Ashwani Kumar Sharma[3], Harry Horsley[4], Klaudia Kloc-Muniak[2], Nor Fadhilah Kamaruzzaman[1,5], Satish Kumar[6] Alexander McFarlane[6], Jem Stach[7], Thomas Bentin[8], , Liam Good[1,2*]

The bactericidal properties of PHMB

PHMB is a widely used antimicrobial biocide and drug (Table 1). Several studies have characterised the antibacterial properties of PHMB against a range of pathogens[1,2]. Here, we reassessed the growth inhibitory and bactericidal properties of PHMB (Supplementary Table 2). As expected we observed potent effects against growth and survival (Supplementary Figure 1).

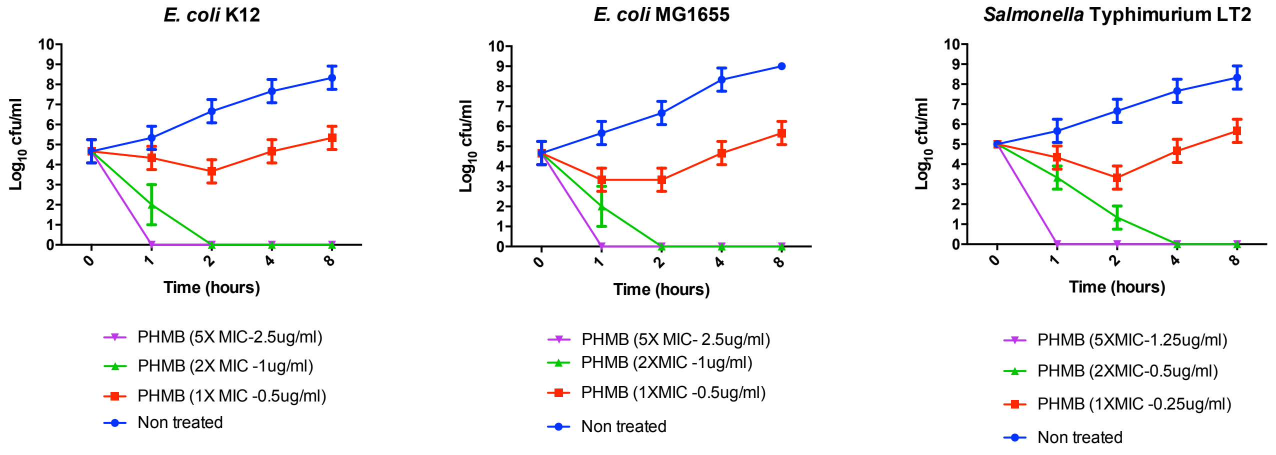

Supplementary Figure 1: PHMB is bactericidal. Survival of *E. coli* and *Salmonella* following exposure to PHMB.

Synthesis of PHMB-FITC conjugates that retain antibacterial activity

PHMB-FITC conjugates were prepared and confirmed by IR spectroscopy (Supplementary Figure 2a,b). Importantly, the conjugates retain antibacterial activity as determined by MIC analysis against *E. coli (strains* K-12 and MG1655) and *S. enterica* serovar Typhimurium LT2

(Supplementary Table 2). Therefore, the results show that the PHMB-FITC preparation is a suitable probe for tracking PHMB cell localization.

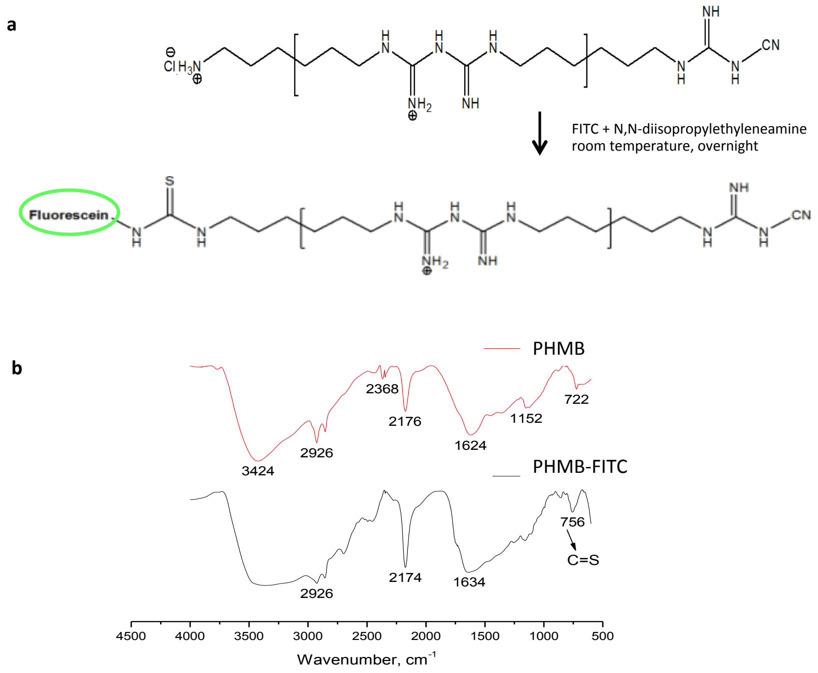

Supplementary Figure 2: PHMB-FITC synthesis scheme. (a) Terminal amino groups in PHMB were chemically conjugated with FITC. (**b**) Formation of a thiourea bond (C=S) between PHMB and FITC was confirmed by infrared spectroscopy.

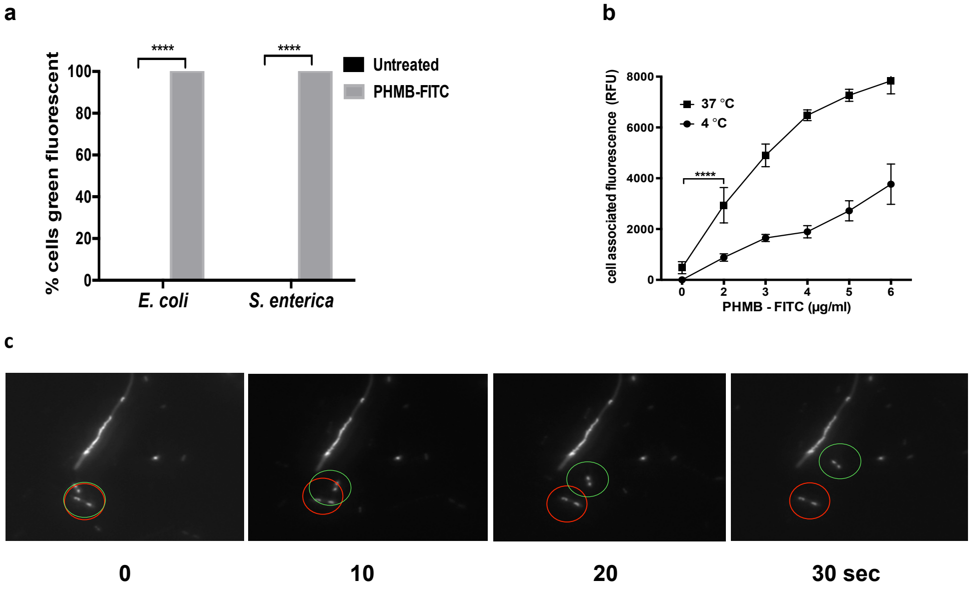

Supplementary Figure 3. Uptake of PHMB-FITC into bacteria (a) Overnight cultures of *E. coli* K-12 and *S. enterica* serovar Typhimurium LT2 were untreated or treated with PHMB-FITC (2 µg/ml) for 90 minutes. The number of cells scoring positive for cell-associated fluorescence was quantified by flow cytometry. ****P < 0.0001. (**b)** Cultures of *E. coli* K-12 (10^8 CFU/ml) were treated with PHMB-FITC (0 – 6 µg/ml) at 4 °C or 37 °C and cell associated fluorescence was measured by fluorimetry (**** P < 0.0001). **(c)** Motility of *E. coli* treated with PHMB. Cultures of *E. coli* K-12 (10^8 CFU/ml) were treated with PHMB (4 µg/ml) at 37 °C for 90 minute, stained with DAPI and observed by using epifluorescence microscopy. Cell movement over time (green circle) is apparent relative to a fixed location (red circle).

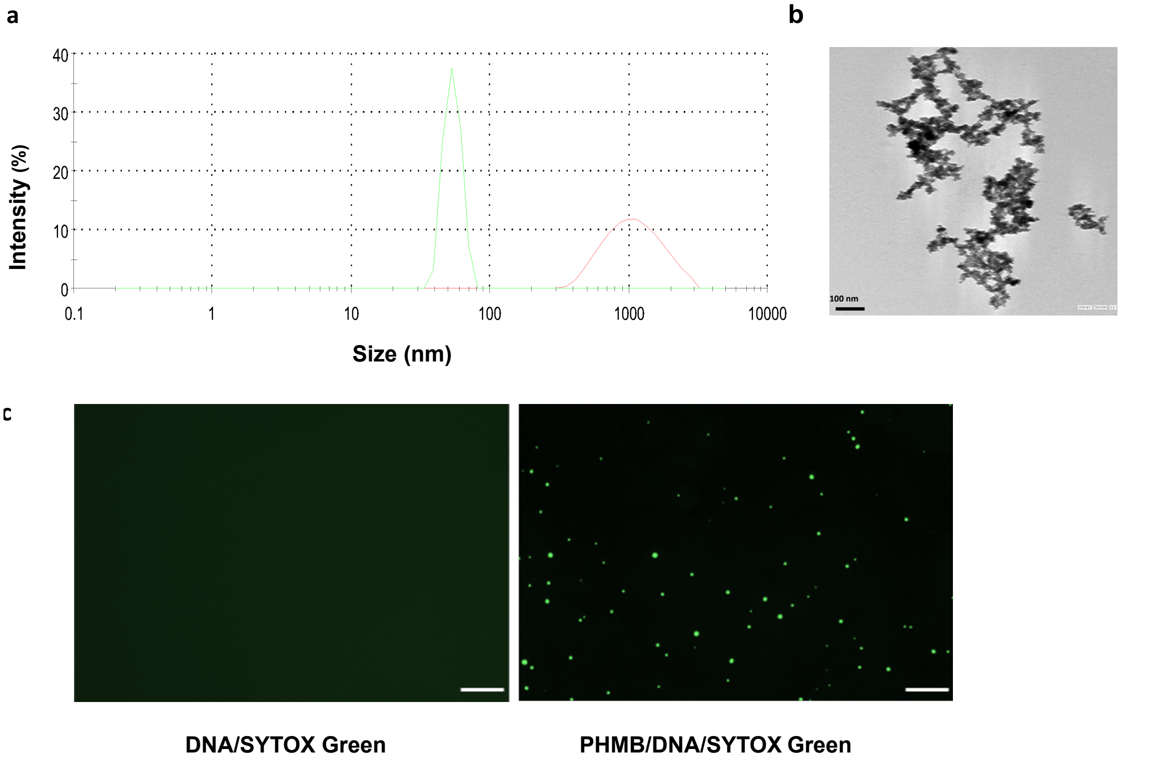

Supplementary Figure 4: PHMB-mediated chromosome condensation *in vitro*. **(a)** Dynamic Light Scattering of PHMB at 2.5 µg/ml (red line) and a mixture of PHMB:DNA at 25 µg/ml:10 µg/ml (green line). The results indicate Z average values (< 100 nm, PDI 0.3). **(b)** Transmission

electron microscopy images of PHMB:DNA nanoparticles. Scale bar = 100 nm. (**c)** PHMB:DNA particle characterization by epifluorescence microscopy. DNA:SYTOX®Green (left) and PHMB:DNA:SYTOX®Green (right) (PHMB:DNA 2.5 μg/ml:1 μg/ml, 100 nM SYTOX®Green in PBS). Scale bars = 10 μm.

PHMB enters HeLa cells without increasing permeability to propidium iodide

To test whether PHMB damages mammalian cell membrane integrity, we used HeLa cells and a propidium iodide (PI) uptake assay. Treatment with PHMB (0 - 4 μg/ml) did not increase HeLa cell staining by PI. Flow cytometry results indicate that >99.8% of cells excluded PI (Supplementary Figure 5a). Therefore, PHMB does not permeabilise mammalian cell membranes to PI at and above concentrations that are inhibitory to bacteria (Supplementary Table 2).

PHMB-FITC fluorescence is pH responsive

To investigate apparent vesicle entrapment of PHMB-FITC and possible localisation within endosomes (see Figure 5a), we exploited the pH responsive fluorescent properties of FITC, which displays a fluorescence drops when the pH is below its pKa of 6.4[3]. We measured the pH effects on PHMB-FITC fluorescence (3.5 μg/ml) by varying the pH from 4 - 7.4. Fluorescence dropped dramatically at reduced pH, confirming quenching of PHMB-FITC at low pH (Supplementary Figure 5b).

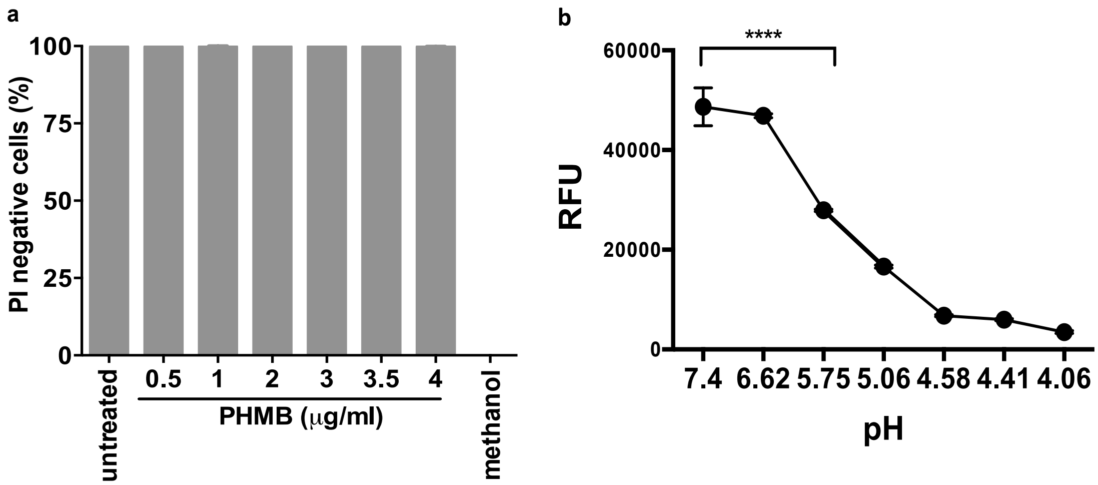

Supplementary Figure 5: PHMB effects on mammalian cells (**a**) Effects of PHMB on propidium iodide entry into mammalian cells. PHMB (0 – 4 µg/ml) was added to HeLa cells and after 2 hours PI (2 µg/ml) was added and cell associated fluorescence was measured by flow cytometry. (**b**) Fluorescence of PHMB-FITC (3.5 µg/ml) in PBS (pH range 4.06 - 7.4), measured by fluorimetry (Excitation 490 nm; Emission 535 nm).

Supplementary Table 1. Examples of products that contain PHMB

**Sector**	**Use**	**Example brand/trade names**
Household	Contact lens cleaner<br>Swimming pool cleanser<br>Cosmetics stabilizer	ReNu® Multipurpose Solution, 0.0001 % w/v, Bausch & Lomb, US.<br>Baquacil™, Revacil®<br>Microcare® MBG™, Microcare® MTB & MTB7
Medicine	Wound therapy<br>Burn wound dressing<br>Eye infection therapy	Prontosan®; B Braun Melsungen AG Ltd, Germany .<br>Kendall™ AMD antimicrobial foam, 0.35 – 0.65 %, w/w, Covidien, USA.<br>Lavasept®, 0.04 %; B Braun Melsungen AG Ltd, Germany.

Supplementary Table 2. MICs of PHMB and PHMB-FITC against bacteria

	**PHMB (µg/ml)**	**PHMB-FITC (µg/ml)**
E. coli K-12	1	1.3
E. coli MG1655	1	1
S. enterica serovar Typhimurium LT2	1	1.3

Supplementary Table 3. MICs against *E. coli* stress response mutants

Strain[a]	Media	Genotype	Description / Function of deleted gene	MIC (µg/ml)	
				PHMB	Nalidixic acid
BW25113	MHB	F-, Δ*(araD-araB)567*, Δ*lacZ4787*(::rrnB-3), *λ*⁻, *rph-1*, Δ*(rhaD-rhaB)568*, *hsdR514*	Keio Parent	2.5	8
JW5060	MHB	BW25113 Δ*bolA::kan*	Induces biofilm formation [4]	2.5	ND
JW2366	MHB	BW25113 Δ*evgA::kan*	Heat resistance [5], acid resistance [6], multidrug efflux [6]	2.5	ND
JW5248	MHB	BW25113 Δ*marA::kan*	Activates multidrug efflux pump [7,8]	2.5	ND
JW3933	MHB	BW25113 Δ*oxyR::kan*	Global oxidative stress regulator [9,10]	2.5	ND
JW2669	MHB	BW25113 Δ*recA::kan*	Induces SOS response [11,12]	2.5	2
JW2755	MHB	BW25113 Δ*relA::kan*	Increases ppGpp alarmone [13]	2.5	ND
JW1555	MHB	BW25113 Δ*relE::kan*	Induces persister formation [14]	2.5	ND
JW5437	MHB	BW25113 Δ*rpoS::kan*	Biofilm formation, general stress response [15,16]	2.5	ND
JW4024	MHB	BW25113 Δ*soxR::kan*	Oxidative stress response [17,18]	2.5	ND
JW4023	MHB	BW25113 Δ*soxS::kan*	Regulates expression of multidrug efflux system [17]	2.5	ND
JW3198	MHB	BW25113 Δ*sspA::kan*	Global regulator, stationary phase stress response [19]	2.5	ND
JW3686	MHB	BW25113 Δ*tnaA::kan*	Biofilm formation [20]	2.5	ND
JW2064	MHB	BW25113 Δ*baeR::kan*	Membrane stress activated multidrug efflux [21,22]	2.5	ND
JW2063	MHB	BW25113 Δ*baeS::kan*	Membrane stress activated multidrug efflux [23,24]	2.5	ND
JW3882	MHB	BW25113 Δ*cpxA::kan*	Multidrug efflux, upregulates peptidoglycan amidase genes [24,25]	2.5	ND
JW3883	MHB	BW25113 Δ*cpxR::kan*	Multidrug efflux, upregulates peptidoglycan amidase genes [23,24]	2.5	ND
JW3367	MHB	BW25113 Δ*envZ::kan*	Osmotic stress response [25,26]	2.5	ND

JW3368	MHB	BW25113 Δ*ompR::kan*	Osmotic stress response [26,27]	2.5	ND
JW1116	MHB	BW25113 Δ*phoP::kan*	Acid resistance, antimicrobial resistance [27,28]	2.5	ND
JW1115	MHB	BW25113 Δ*phoQ::kan*	Acid resistance, antimicrobial resistance [28,29]	2.5	ND
JW2205	MHB	BW25113 Δ*rcsB::kan*	Biofilm formation, antimicrobial resistance [29,30]	2.5	ND
JW5917	MHB	BW25113 Δ*rcsC::kan*	Biofilm formation, antimicrobial resistance [29,30]	2.5	ND
JW2204	MHB	BW25113 Δ*rcsD::kan*	Biofilm formation, antimicrobial resistance [29,30]	2.5	ND
ASKA JW2669	MHB	*Cm;*$lacI^q$*, pCA24N::*$recA^+$	Overexpression of *recA*	2.5	8
	MHB IPTG (1 mM) Chl (30 µg/ml)			2.5	64
K-12[*]	MHB	F^- λ^-	*E. coli* wild-type	1.25	8
AB2474[*]	MHB	*lexA1*(Ind^-)	Non-cleavable LexA repressor protein; defective in SOS induction, UV sensitive [31,32]	0.625	16

[a] Strains were obtained from the Keio[2] and ASKA[3] libraries. K-12 was from CGSC. K-12 was obtained from CGSC (#5073). AB2474 is a K-12 derivative; for both strains, the MIC for ampicillin was 12.5 µg/ml, and for the MIC for kanamycin was 1.6 µg/ml for AB2474 and 3.2 µg/ml for K-12.

Supplementary Table 4. MIC and FICI values in diverse bacteria

Species	Strain (Source)	AT%	GC%	PHMB MIC (µg/ml)	Hoechst MIC (µg/ml)	PHMB: Hoechst FICI	trimethoprim MIC (µg/ml)	PHMB: trimethoprim FICI	triclosan MIC (µg/ml)	PHMB: triclosan FICI
Bacillus megaterium	DSM 319 (DSMZ)	62	38	10	5	1.5	1	0.8	1	1.1
Bacillus subtilus	BsCPH1 (JS lab.)	54	46	5	2.5	1.25	0.5	0.8	1	0.8
Escherichia coli	MG1655 (CGSC#5073)	49	50	2	5	3.0	0.5	1.0	2	1.0
Micrococcus luteus	M9.25 (JS lab.)	25	75	10	2.5	1.0	2	0.6	1	0.75
Mycobacterium smegmatis	MC2 155 (*)	32	67	12	5	1.75	2	1.0	2	0.8
Pseudomonas aeruginosa	PA01 (JS lab.)	34	65	10	12	1.25	4	1.0	4	1.0
Salmonella enterica typh.	LT2 (JS lab.)	48	52	4	6	1.5	3	0.75	1	0.9
Staphlylococcus epidermidis	SERVC1 (JS lab.)	68	32	1	5	1.25	1	0.8	1	1.2
Staphylococcus aureus	RN4220 (*)	67	33	2		2.0	3	0.75	0.5	0.6
Streptomyces griseus	SGJS1 (JS lab.)	28	72	5	5	1.25	1	1.2	1	1.0

*Ms strain MC2 155 was obtained from WR Jacobs, AEMS, Bronx, NY, and Sa strain RN4220 was obtained from Staffan Arvidsson, KI, Stockholm.

References:

1. Muller, G., Koburger, T. & Kramer, A. Interaction of polyhexamethylene biguanide hydrochloride (PHMB) with phosphatidylcholine containing o/w emulsion and consequences for microbicidal efficacy and cytotoxicity. *Chem Biol Interact* **201,** 58–64 (2013).
2. Gilbert, P., Das, J. R., Jones, M. V & Allison, D. G. Assessment of resistance towards biocides following the attachment of micro-organisms to, and growth on, surfaces. *J. Appl. Microbiol.* **91,** 248–54 (2001).
3. Murphy, R. F., Powers, S. & Cantor, C. R. Endosome pH measured in single cells by dual fluorescence flow cytometry: rapid acidification of insulin to pH 6. *J. Cell Biol.* **98,** 1757–62 (1984).
